# Manipulation and quantification of microtubule lattice integrity

**DOI:** 10.1242/bio.025320

**Published:** 2017-06-29

**Authors:** Taylor A. Reid, Courtney Coombes, Melissa K. Gardner

**Affiliations:** Department of Genetics, Cell Biology, and Development, University of Minnesota, Minneapolis, MN 55455, USA

**Keywords:** Microtubule, Taxol, Structure, Lattice, GMPCPP

## Abstract

Microtubules are structural polymers that participate in a wide range of cellular functions. The addition and loss of tubulin subunits allows the microtubule to grow and shorten, as well as to develop and repair defects and gaps in its cylindrical lattice. These lattice defects act to modulate the interactions of microtubules with molecular motors and other microtubule-associated proteins. Therefore, tools to control and measure microtubule lattice structure will be invaluable for developing a quantitative understanding of how the structural state of the microtubule lattice may regulate its interactions with other proteins. In this work, we manipulated the lattice integrity of *in vitro* microtubules to create pools of microtubules with common nucleotide states, but with variations in structural states. We then developed a series of novel semi-automated analysis tools for both fluorescence and electron microscopy experiments to quantify the type and severity of alterations in microtubule lattice integrity. These techniques will enable new investigations that explore the role of microtubule lattice structure in interactions with microtubule-associated proteins.

## Abstract (precis)

**Summary:** In this work, new tools were developed to manipulate and quantitatively measure the lattice structural state of microtubules for three nucleotide states, which were assessed using electron and fluorescence microscopy.

## INTRODUCTION

Microtubules are long, hollow tubes that act as important structural and signaling components inside cells. Microtubules are typically closed tubes that are formed by 13 laterally associated individual protofilaments, each of which is composed of αβ-tubulin heterodimers that are stacked end-to-end (Zhang et al., 2015; Wang and Nogales, 2005). However, while this regular, stacked αβ heterodimer arrangement of microtubules is widely conserved, electron microscopy studies have revealed the presence of a wide range of microtubule lattice structures and irregularities. For example, cryo-electron microscopy studies have revealed that the lattice structures near to growing microtubule ends are frequently characterized by flattened, open sheets, rather than closed tubes (Chrétien et al., 1995; Guesdon et al., 2016). Further, variations in the number of individual protofilaments have been observed both within a microtubule (Vitre et al., 2008; Doodhi et al., 2016) and between microtubules that are nucleated under different conditions (Vitre et al., 2008; Moores et al., 2012; Wade and Chrétien, 1993; des Georges et al., 2008), leading to heterogeneity and defects in the microtubule lattice. It has been recently reported that hydrolysis of the β-tubulin subunit within the microtubule lattice leads to overall ‘compaction’ of the microtubule lattice (Alushin et al., 2014), likely leading to structural heterogeneity within the microtubule lattice. Finally, a range of microtubule-targeting drugs have been reported to alter the large-scale microtubule structure, introducing heterogeneity and defects into the microtubule lattice (Díaz et al., 1998; Doodhi et al., 2016; Kellogg et al., 2017).

Importantly, recent work has uncovered links between microtubule lattice integrity and the efficiency of kinesin-based transport (Liang et al., 2016), katanin-mediated microtubule severing (Davis et al., 2002), microtubule destabilization by Stathmin (Gupta et al., 2013) and tubulin acetylation in microtubules (Coombes et al., 2016). Similarly, disruption of the closed microtubule lattice structure near to the growing microtubule end hints that microtubule tip-tracking proteins could recognize this configuration to facilitate tip tracking (Guesdon et al., 2016; Bechstedt and Brouhard, 2012; Bechstedt et al., 2014). Thus, microtubule lattice integrity may significantly impact a variety of microtubule-associated cellular processes. For this reason, tools are required both to systematically manipulate microtubule lattice integrity in an *in vitro* setting, and also to quantitatively assess the associated microtubule lattice structure.

However, methods to systematically generate *in vitro* microtubule pools with common nucleotide states, but with differing states of lattice structural integrity, have not been described. These microtubule pools would be invaluable for assessing the contribution of microtubule lattice integrity to various microtubule-associated cellular processes. Similarly, while variations in lattice integrity have been observed using electron microscopy (Coombes et al., 2016), methods to quantify and describe these variations would be a useful contribution to this newly developing field of study.

In this work, we describe new methods for generating pools of *in vitro* microtubules with common nucleotide states, but with differing degrees and types of disruptions in lattice integrity. In addition, we have developed new analytical tools for quantifying these microtubule structural states through (1) a semi-automated image analysis platform for Electron Microscopy (EM) images, and (2) experiments and a semi-automated analysis method using Total Internal Reflection Fluorescence (TIRF) microscopy. Through our new quantitative tools, we found that the growth and storage conditions for *in vitro* microtubules had a strong impact on the lattice structural integrity of the microtubules. These results have implications that should be considered when investigating the interactions of microtubules with a range of microtubule-associated proteins, such as molecular motors, microtubule tip-tracking proteins, post-translational modification enzymes, and microtubule severing enzymes.

## MATERIALS, METHODS AND RESULTS

### Method: GDP-Tubulin microtubule pools with potential variations in lattice structural states

**Fig. 1.**
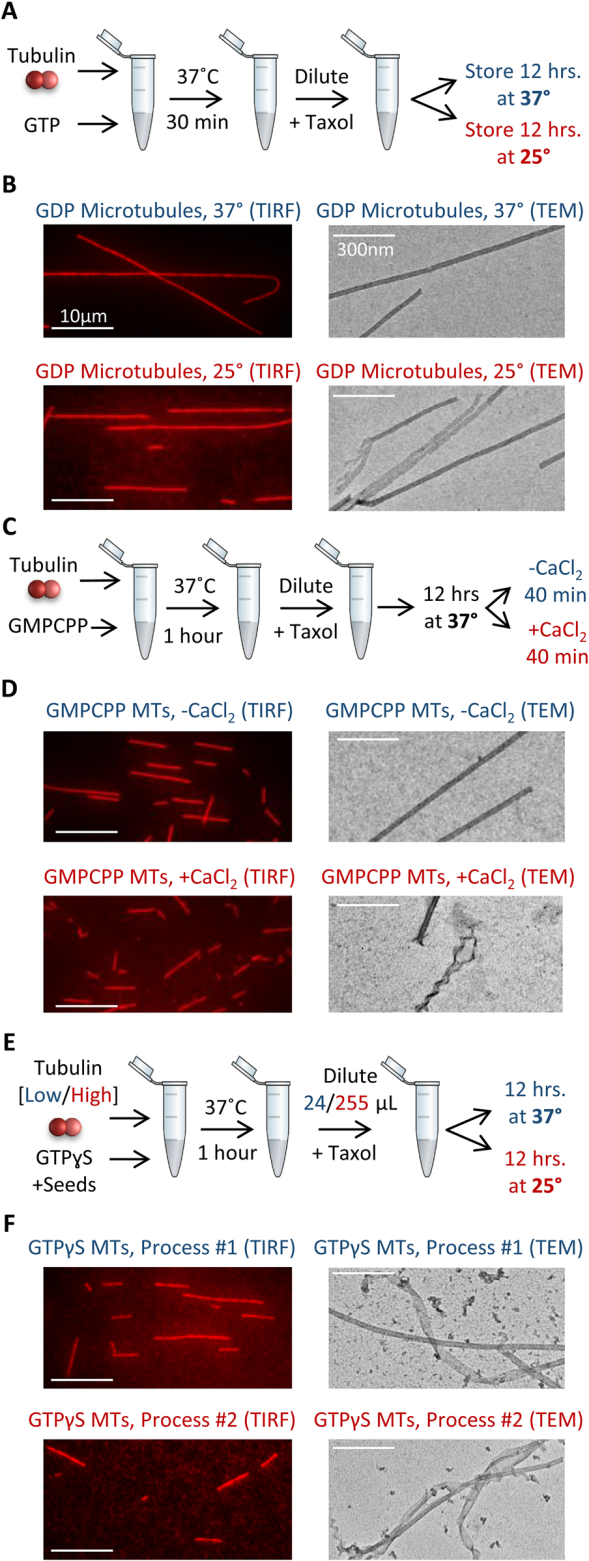
**Microtubule nucleotide pool preparation.** (A) Growth protocol and storage conditions for GDP microtubules. (B) TIRF images of 37°C storage GDP microtubules (left, top), and 25°C storage GDP microtubules (left, bottom), as well as EM images for each (right). (C) Growth protocol, storage conditions, and CaCl_2_ treatment protocol for GMPCPP microtubules. (D) TIRF images of GMPCPP microtubules, both untreated (left, top), and CaCl_2_ treated (left, bottom), as well as EM images for each (right). (E) Growth protocol and storage conditions for GTPγS microtubules. (F) TIRF images of GTPγS microtubules prepared and stored according to Process #1 (left, top), and Process #2 (left, bottom), as well as EM images for each (right). For all TEM images, microtubules were chosen to highlight the differences in structure that were observed, though many microtubules from the altered preparations (red) resemble those from the control preparation (blue). Images to represent the average condition are shown in Fig. 3.

We first developed a method to generate stable pools of *in vitro* microtubules with a common GDP-tubulin nucleotide state, but with differing degrees and types of disruptions in lattice integrity. To prepare GDP-tubulin microtubules, a mixture composed of 33 µM tubulin (see Supplementary Materials and Methods) (25% rhodamine-labeled, 75% unlabeled), 1 mM GTP, 4 mM MgCl_2_, and 4% DMSO was prepared and kept on ice for 5 min, and then incubated at 37°C for 30 min. Following incubation, 10 μl of the microtubule mixture was diluted in a single step into 990 μl warm, 10 μM Taxol solution (Sigma-Aldrich) in Brb80 (80 mM PIPES pH 6.9, 1 mM EGTA, 1 mM MgCl_2_) (Fig. 1A).

To potentially manipulate the microtubule structural states, and to ensure that the microtubules were fully hydrolyzed into GDP-tubulin, the microtubule solution was then separated into two tubes for overnight storage. One tube was stored overnight at 37°C, while the second tube was stored overnight at 25°C (Fig. 1A). Here, we predicted that the 37°C storage condition could potentially promote more efficient lattice repair of the microtubules, as compared to the solution stored at 25°C. This idea was supported by the observation that microtubules stored at 37°C were longer than those stored overnight at 25°C (Fig. 1B, left; quantitative length analysis Fig. S1A; *P*=2×10^−9^, *t*-test), and preliminary EM images hinted that there were also differences in lattice structural integrity as a result of these alternative storage conditions (Fig. 1B, right).

We note that in order to generate stabilized GDP-tubulin microtubules, Taxol was added to the microtubule solution prior to overnight storage, replicating a widely used approach in microtubule research. Because Taxol itself has been reported to have an effect on microtubule structure (Kellogg et al., 2017; Díaz et al., 1998), the 25°C and 37°C storage condition microtubule mixture tubes were identically treated with Taxol prior to storage. Therefore, any observed changes in microtubule structure after overnight storage would be independent of the effect of the initial Taxol treatment itself.

### Method: GMPCPP-tubulin microtubule pools with potential variations in lattice structural states

To prepare stabilized GTP-tubulin microtubules, we used the slow-hydrolyzing analogue, GMPCPP. Because GMPCPP microtubules are very stable, and self-nucleate at low free tubulin concentrations, lattice integrity disruptions of GMPCPP microtubules were accomplished by post-assembly treatment with CaCl_2_, which disassembles GMPCPP microtubules into protofilamentous structures (Gupta et al., 2013), and when used at an intermediary degree of exposure, results in microtubules in varying stages of damage and disassembly (Coombes et al., 2016).

To make stabilized GMPCPP microtubules, 3.9 µM tubulin (25% rhodamine-labeled, 75% unlabeled) and 1 mM GMPCPP in Brb80 was mixed and kept on ice for 5 min, then incubated at 37°C for 1 h. Following incubation, the microtubules were diluted into 400 µl warm Brb80, and 350 µl of this dilution was spun down in an air-driven ultracentrifuge (Beckman-Coulter, Indianapolis, IN, USA) at 20 psi for 5 min. The supernatant was discarded, and the pellet resuspended into 400 µl warm Brb80 with 10 μM Taxol to further stabilize the microtubules. Then, the microtubule mixture was separated into two batches. One batch remained untreated, and the second batch was incubated in a 0.04 M final concentration of CaCl_2_ for 40 min at 37°C immediately prior to use in microscopy assays (Fig. 1C). While there was no length difference in these microtubule preparations using TIRF microscopy (Fig. 1D, left; quantitative length analysis Fig. S1C; *P*=0.26, *t*-test), EM images demonstrated occasional disruptions in microtubule lattice integrity, and protofilament unwinding, for the CaCl_2_-treated microtubules (Fig. 1D, right).

Similar to the GDP microtubule nucleotide preparations, Taxol was added to the microtubule solution to further stabilize the microtubules, and especially to preserve the CaCl_2_-treated GMPCPP microtubules. However, both untreated and CaCl_2_-treated microtubules were identically mixed with Taxol prior to imaging. Therefore, any observed changes in microtubule structure between untreated and CaCl_2_-treated microtubules would be independent of the effect of Taxol treatment.

### Method: GTPγS-tubulin microtubule pools with potential variations in lattice structural states

Finally, we prepared stabilized GTP-tubulin microtubules utilizing the GTP analogue GTPγS. Here, two different preparation methods were employed. In Process #1, a mixture composed of 12 µM tubulin (25% rhodamine-labeled, 75% unlabeled), 50 mM KCl, 10 mM DTT, 0.1 mg/ml Casein, 4 mM GTPγS and unlabeled GMPCPP ‘seed’ microtubules was prepared and incubated at 37°C for 1 h. After 1 h, 10 μl of the microtubule mixture was diluted into 24 μl warm Brb80 with 50 mM KCl, 10 mM DTT, 0.1 mg/ml Casein and 10 μM Taxol, and stored overnight at 37°C (Fig. 1E). We predicted that the Process #1 protocol would maximize the possibility of producing GTPγS microtubules with intact lattice structures, because (1) the relatively low tubulin concentration used in the initial microtubule assembly may promote a more ordered assembly process (Gardner et al., 2011), and (2) storage of Taxol-stabilized GTPγS microtubules at 37°C may promote more efficient lattice defect repair of the microtubules, as described above.

We then used an alternative process, Process #2, to produce GTPγS microtubules with potentially more disrupted lattice structures. This process was identical to Process #1, except that (1) the tubulin concentration used in the initial microtubule assembly was 25.5 μM rather than 12 μM, since we predicted that a higher free tubulin concentration may promote a more rapid, and thus less ordered, more defect-prone assembly process (Gardner et al., 2011), and (2), after 1 h of assembly, 10 μl of the microtubule mixture was diluted into 255 μl warm Brb80 with 50 mM KCl, 10 mM DTT, 0.1 mg/ml Casein, and 10 μM Taxol (as compared to 24 μl as described above), and, (3) the mixture was stored overnight at 25°C (in contrast to 37°C as above) (Fig. 1E). Here, we predicted that by reducing the residual free tubulin concentration during storage, and by storing the microtubules at a lower temperature, this would discourage any lattice defect repair of the GTPγS microtubules. Indeed, microtubules stored at 37°C, and with a higher residual free tubulin concentration (Process #1 above), were substantially longer than those stored overnight at 25°C at a lower residual free tubulin concentration (Process #2) (Fig. 1F, left; quantitative length analysis Fig. S1B; *P*=2×10^−16^, *t*-test), suggesting that polymerization and repair may have occurred during storage. Preliminary EM images hinted that both preparations had some degree of disruptions in large-scale microtubule lattice integrity, with Process #2 having more frequent disruptions (Fig. 1F, right).

Similar to the other microtubule preparations, we note that in order to generate stabilized GTPγS-tubulin microtubules, Taxol was added to the microtubule solution prior to overnight storage, again replicating a widely used approach in microtubule research. Because Taxol itself has been reported to have an effect on microtubule structure (Kellogg et al., 2017; Díaz et al., 1998), the Process #1 and Process #2 microtubule mixture tubes were identically treated with Taxol prior to storage. Therefore, any observed changes in microtubule structure between Process #1 and Process #2 would be independent of the effect of Taxol treatment.

### Method: Quantitative lattice structural characterization tool for EM

We then collected images of each microtubule preparation using Transmission Electron Microscopy (TEM), and analyzed the images for potential structural disruptions using a newly developed semi-automated analysis tool. It should be noted that the negative stain TEM method used here provides a simple method for comparative analysis of our microtubule pools, especially since both pools of microtubules in each nucleotide case were identically prepared and imaged with TEM over multiple trials. However, our new automated tool for quantitative structural characterization of microtubules as is described below would be equally applicable to cryo-electron microscopy, a method that may allow for improved preservation of microtubule structure.

For TEM imaging, microtubules were prepared identically to those as described above. A drop of the mixture was then placed on a 300-mesh carbon-coated copper grid for 1 min. After 1 min, the grid was stained with 1% uranyl acetate for 1 min. The stain was then wicked away with filter paper and the grid was left to dry and then stored. Specimens were imaged using a Technai Spirit BioTWIN transmission electron microscope (FEI, Thermo Fisher Scientific). All images were acquired at 18.5k× magnification (pixel size, 1 nm), 2048×2048 image size, and saved to a lossless image format.

**Fig. 2.**
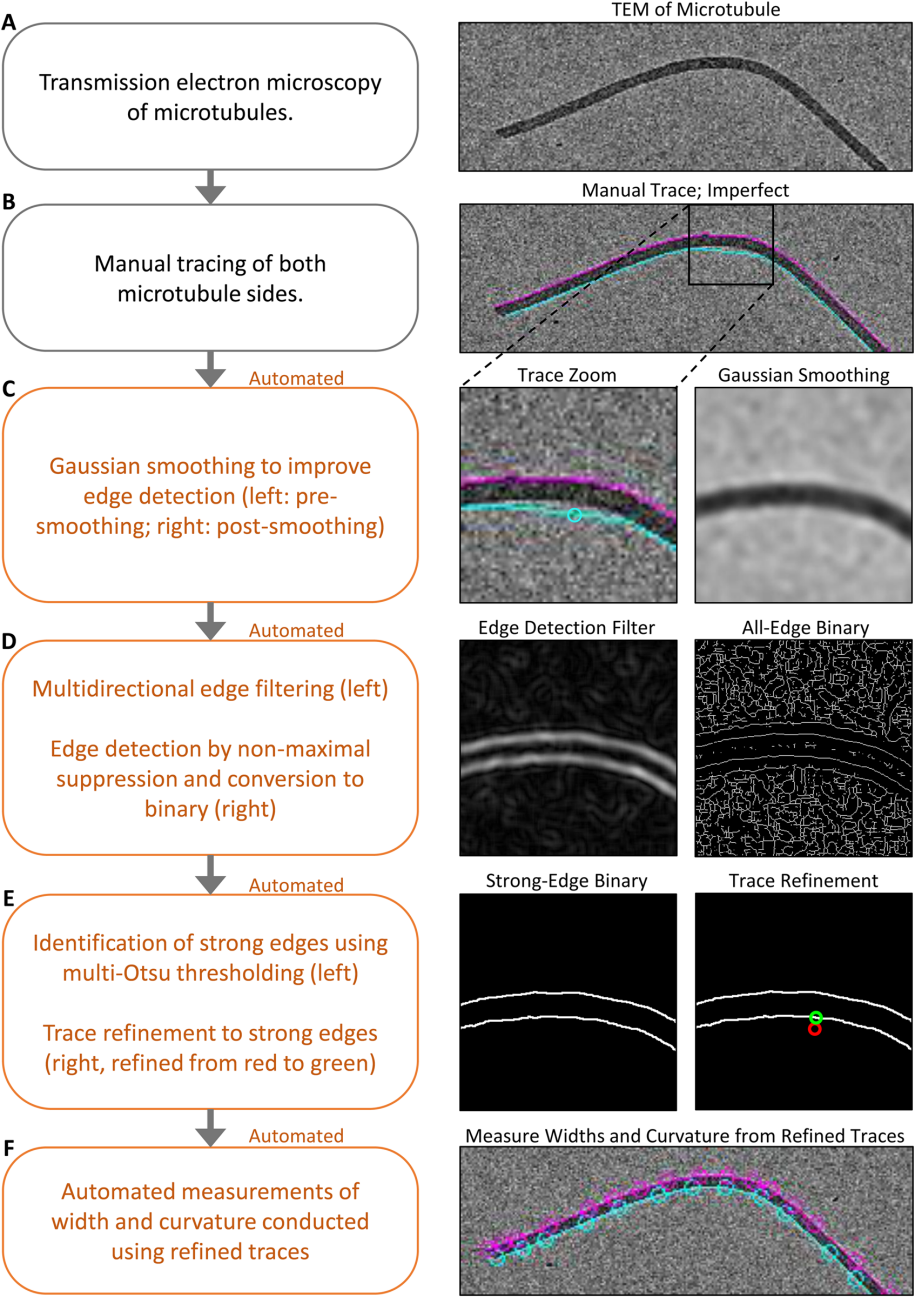
**Automated quantification of large-scale microtubule lattice integrity.** Description (left) and example visualization (right) of the automated EM quantification method. The boxed region in B (right) is shown enlarged in C to E (right).

Analysis of the EM images was performed using a novel custom MATLAB (MathWorks) script (see Supplementary Materials and Methods). First, microtubules in the EM images were traced manually using connected line segments (Fig. 2A,B). We note that the segment size resulting from manual tracing was dependent on the degree of curvature, with higher curvature leading to shorter segments. The segments were refined using an automated algorithm to reduce human error or bias (Fig. 2C-E). This automated refinement involved first smoothing the image to improve edge detection (Fig. 2C), followed by the use of an edge filter and nonmaximal suppression, which is an intensity based thinning technique to identify the center of the edges (Fig. 2D). Finally, the ‘strong’, high intensity, microtubule edges were identified using a multi-level implementation of the Otsu thresholding algorithm with which the manual edge traces could be refined (Fig. 2E). From the refined microtubule traces, microtubule width (*W*) and curvature (*C*) were measured automatically (Fig. 2F) (for details of trace segmentation and midline calculation see Supplementary Materials and Methods).

**Fig. 3.**
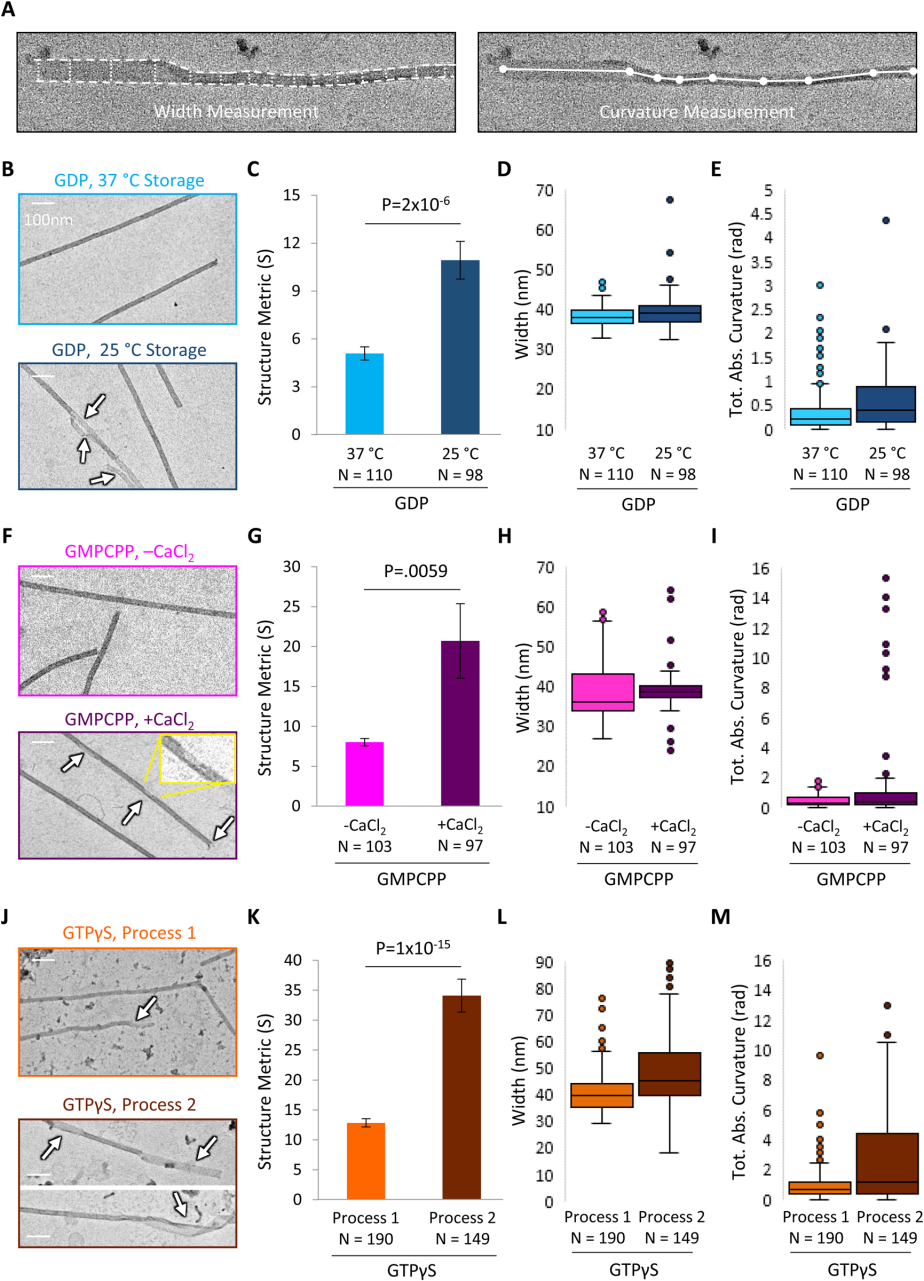
**Large-scale lattice integrity is shifted within microtubule pools.** (A) Visualization of EM quantification method. Left: width measurement in the Structure Metric. Right: curvature measurement in the Structure Metric. (B) Sample EM images of 37°C storage GDP microtubules (top) and 25°C storage GDP microtubules (bottom; arrows indicate structural disruptions). (C) Structure Metric is increased at 25°C storage, suggesting that large-scale microtubule structure is disrupted. (D,E) Width measurements and curvature measurements contribute to the Structure Metric. (F) Sample EM images of untreated GMPCPP microtubules (top) and CaCl_2_ treated microtubules (bottom; arrows indicate structural disruptions, inset is enlargement). Scale bars: 100 nm. Structure Metric is increased with CaCl_2_ treatment (bottom), suggesting that large-scale microtubule structure is disrupted. (H,I) Width and curvature measurements contribute to structure metric. (J) Sample EM images of GTPγS microtubules prepared and stored using Process #1 (top) and Process #2 (bottom; arrows indicate disrupted structure). Scale bars: 100 nm. Structure Metric is increased with Process #2, suggesting that large-scale microtubule structure is further disrupted with Process #2 as compared to Process #1. (L,M) Width and curvature measurements contribute to the overall Structure Metric. The bar graphs in C, G and K show mean±s.e.m.; *P*-values were calculated from *t*-test of independent means.

The width and curvature metrics were then combined to calculate an overall ‘Structure Metric’ (*S*), which provides a quantitative measure of the morphology of microtubule lattice. To do this, the total absolute curvature was calculated by summing the absolute value of curvature for each segment of an individual microtubule midline [Fig. 3A, left; (*C_Total_*)]. Then, a microtubule ‘width deviation’ metric was automatically calculated by measuring the width of the microtubule for each segment [Fig. 3A, right, (*W*)], and then by subtracting the width of a typical intact microtubule (*W_Expected_*) in pixels as measured based on typical intact microtubules in the images. The absolute value of this width deviation was summed across the whole microtubule, normalized to microtubule length, and used as the width deviation metric (|*W*−*W_Expected_*|). The final Structure Metric (*S*) was then calculated by summing the width deviation and curvature metrics, each respectively normalized by the parameters *N_W_* and *N_C_* to provide approximately equal weight of curvature and width to the final score, as follows:(1)
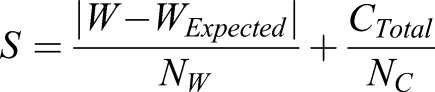

The values of *N_W_*  and *N_C_* were set to 2 and 0.1 respectively, such that a deviation in width of 2 pixels was weighted equally with a total absolute curvature of 0.1 radians. These values were chosen according to the scale of variation observed in width and curvature in order that each contributed approximately 50% to the final overall Structure Metric (*S*). We note that a larger value of the EM Structure Metric (*S*) would reflect more frequent or more drastic morphological structural disruptions in microtubule lattice integrity, such as bends, partial tubes, and open sheet-like regions, which would tend to increase both microtubule width and curvature. Smaller disruptions in microtubule lattice integrity, such as defects or holes in the lattice, would be less efficiently detected by this measure.

### Results: Microtubule pools have alterations in lattice structural integrity as measured by EM

The automated MATLAB code was then applied to analyze EM images for each pooled batch of microtubules. By using a common Structure Metric based on microtubule width and curvature (*S*, Eqn 1), the large-scale structural integrity of microtubule preparations could be compared between pools of microtubules with different preparations. Significance was assessed using two-tailed Student's *t*-test of independent means. All imaged microtubules were included in the analysis regardless of apparent structural condition. Images in Fig. 3 were chosen to match the average structure metric of each condition.

First, the lattice integrity of the GDP microtubules was evaluated. Qualitatively, the pool of 37°C storage-condition GDP microtubules was characterized in the EM images by straight edges and uniform widths (Fig. 3B, top). In contrast, the 25°C storage-condition microtubule pool appeared to have more frequent bends along the length of the microtubule, and higher variability in width along the microtubule length, frequently coincident with regions of reduced intensity (Fig. 3B, bottom). These disruptions may be associated with an open, sheet-like, or incomplete tubes. We then used our automated analysis tool to measure the Structure Metric (S) of numerous microtubules in each pool. We found that there was a significant increase in the Structure Metric for the 25°C storage-condition pool of GDP microtubules as compared to the 37°C storage-condition (Fig. 3C; *P*=2×10^−6^, *t*-test), and that this increase was due to shifts towards larger width variation and a larger curvature of the microtubules (Fig. 3D,E), suggesting that the lower temperature storage condition reduced the lattice structural integrity (i.e. led to increased incidence of structural disruptions) of the Taxol-stabilized GDP microtubules. Note that Fig. 3D, and corresponding figures for the other nucleotides (Fig. 3H,L), show the average microtubule width, although the structure metric is a function of cumulative width deviation.

Then, the lattice integrity of the GMPCPP microtubules was evaluated. Qualitatively, the GMPCPP microtubules without CaCl_2_ treatment appeared predominantly straight and uniform, similar to the intact GDP microtubules, (Fig. 3F, top). In contrast, the GMPCPP microtubules with CaCl_2_ treatment appeared to have more disruptions (Fig. 3F, bottom), and occasionally exhibited a characteristic feature of ‘unraveled’ filamentous regions (Fig. 1F). Quantitatively, we observed a significant increase in the Structure Metric (*S*) for the calcium-treated GMPCPP microtubules (Fig. 3G, center; *P*=5.9×10^−3^, *t*-test). This increase appeared to be due largely to the increased curvature of ribbon-like microtubule structures (Fig. 3I and Fig. 1F), since CaCl_2_ treatment narrowed the typical width distribution (Fig. 3H), likely by generating gaps or holes in the lattice, and by selectively depolymerizing the more unstable, wider, open structures. This suggests that treatment with CaCl_2_ acted to disrupt the lattice structural integrity of GMPCPP microtubules. The CaCl_2_-treated microtubules had a high variance in Structure Metric, as some of the microtubules had very structurally distinct unraveled regions (Fig. 1F).

Finally, the lattice structural state of the GTPγS microtubules was evaluated. The pools of GTPγS microtubules generated using Process #2 appeared qualitatively more curved, and of less uniform width, than the GTPγS microtubules from Process #1 (Fig. 3J). This observation was quantitatively confirmed by evaluating the Structure Metric score: there was a significant increase in the Structure Metric value for the GTPγS microtubules produced and stored via Process #2 as compared to Process #1 (Fig. 3K; *P*=1×10^−15^, *t*-test). This increase came about by concurrent shifts towards larger widths and higher curvature for the Process #2 GTPγS microtubules as compared to Process #1 (Fig. 3L,M). These results suggest that the GTPγS microtubules produced by Process #2 tended to have more frequent regions with open sheets and partial tubes relative to those produced by Process #1.

While the preparation protocols for microtubules using the three different nucleotides were distinct, each of these protocols reflect commonly used methods for producing stable *in vitro* microtubules. As such, our new analysis method highlights structural differences in the microtubule lattice that are produced when these protocols are used in a typical laboratory setting. In particular, we note that even the intact microtubules from Process #1 for the GTPγS microtubules had a much higher structure metric (∼13), and thus substantially lower structural integrity, than the microtubules produced from either of the common base protocols for the GDP and GMPCPP microtubules (∼4 and ∼7, respectively).

Importantly, we have described methods that allowed us to shift the lattice structural integrity within a given nucleotide pool of stable microtubules. Quantification of microtubule EM images suggested that the large-scale lattice structural integrity of a microtubule, as assessed by its width and curvature, is tunable for Taxol-stabilized GDP, GMPCPP and GTPγS microtubules. The ability to shift the large-scale structural integrity of microtubules within a common nucleotide state will allow for new studies that directly examine the effect of microtubule structural state on steady-state binding, mobility, and on/off kinetics of microtubule-associated proteins.

### Method: Lattice structural characterization by TIRF reporter assay

The automated EM quantification tool described above provided a method to characterize lattice structural changes in *in-vitro* stabilized microtubules. However, this EM quantification method was not efficient in characterizing smaller, submicrotubule-scale disruptions in microtubule lattice integrity, such as gaps or holes. For such an analysis, we developed an alternative automated method.

**Fig. 4.**
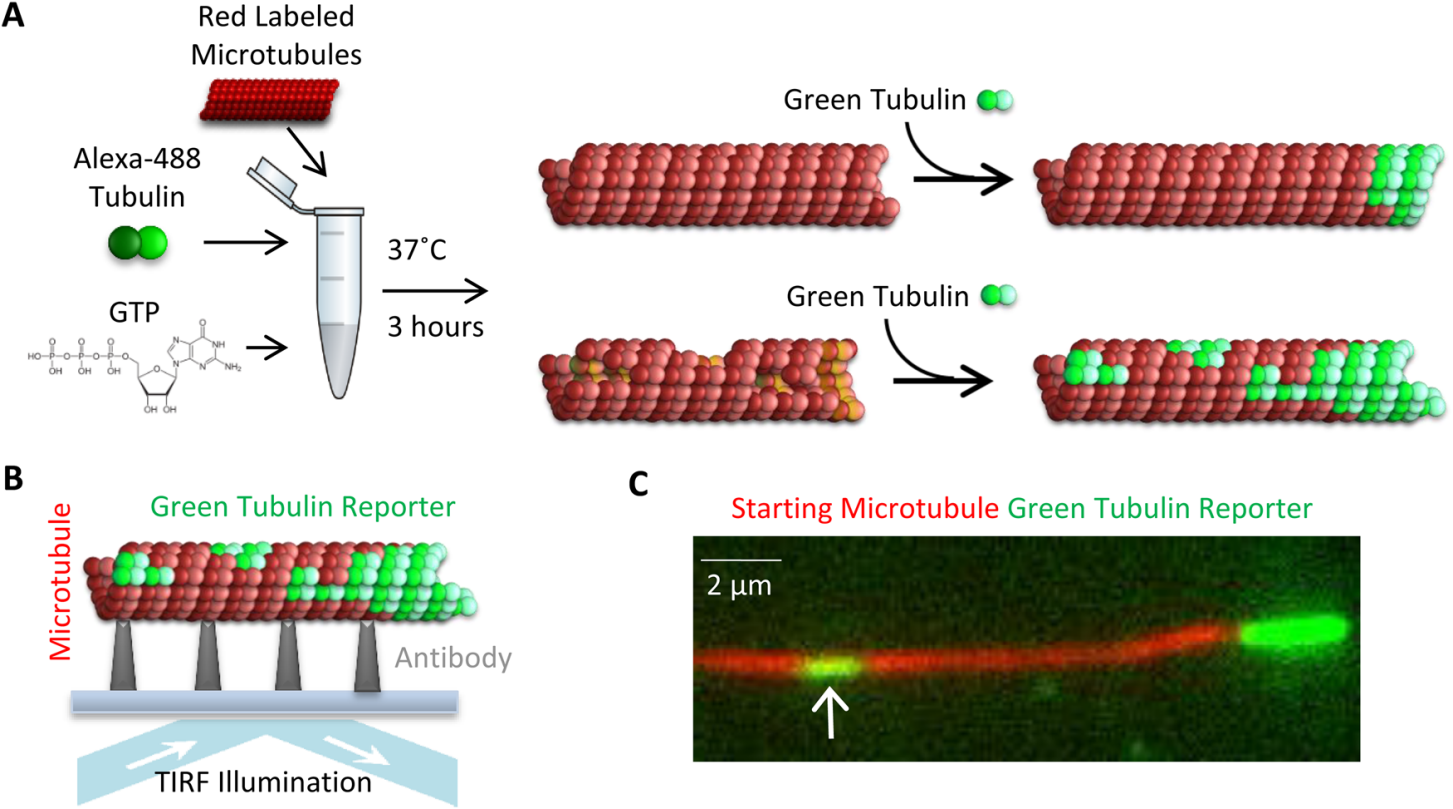
**Experimental reporter assay.** (A) Depiction of the experimental reporter assay: green reporter tubulin incorporates at microtubule plus ends, and at gaps and defects in the lattice through a repair process. The amount of lattice-incorporated green reporter tubulin is expected to be higher for more disrupted microtubule structures (right, bottom), and lower for more intact lattice structure (right, top), but green extensions at microtubule plus-ends will be observed in both cases. (B) Depiction of TIRF imaging process. (C) Example image, showing a microtubule with a green tubulin extension at the plus end (right), with a gap that has been filled by green reporter tubulin (left, white arrow).

Recent work by Schaedel et al. (2015) demonstrated that new tubulin could be incorporated into defects or gaps in the microtubule lattice. Based on this result, we developed a TIRF ‘reporter’ assay, which allowed us to quantitatively probe the structural integrity of our microtubule pools using fluorescence microscopy. The experimental portion of our reporter assay was completed as follows. First, each red-labelled microtubule pool (as described above) was incubated with green-labelled ‘reporter’ tubulin. To do this, the final microtubule preparations as described above were spun down in an air-driven ultracentrifuge at 20 psi for 5 min, resuspended in 50 μl of ‘reporter’ solution [1.5 μM 66% Alexa Fluor 488 (Thermo Fisher Scientific)-labelled tubulin, 1 mM MgCl_2_, 250 μM GTP, and 10 μM taxol in Brb80], and then incubated for 3 h at 37°C (Fig. 4A, left). This microtubule solution was then introduced into an imaging chamber, after which between 30 s and 3 min were allowed for the microtubules to adhere to the imaging coverslip, and the solution was subsequently replaced with warm imaging buffer (see Materials and Methods). The microtubules were then imaged at 488 nm and 561 nm wavelengths (Fig. 4B).

Over the course of the incubation period, the green reporter tubulin incorporated as normal microtubule growth at the plus ends of microtubules (Fig. 4C, right), but was also occasionally incorporated along the length of the microtubule (Fig. 4C, left, white arrow). Here, we expected that microtubules with more gaps, holes, or other lattice defects would lead to an increased occurrence of green reporter patches along the length of the microtubule due to new reporter tubulin incorporation into the lattice (Fig. 4A, right, bottom). In contrast, a perfectly intact microtubule lattice would only have green reporter tubulin incorporation extending beyond the red-labelled lattice at its plus-end, due to normal microtubule end assembly (Fig. 4A, right, top).

### Method: Lattice structural characterization by TIRF reporter assay–quantitative analysis

**Fig. 5.**
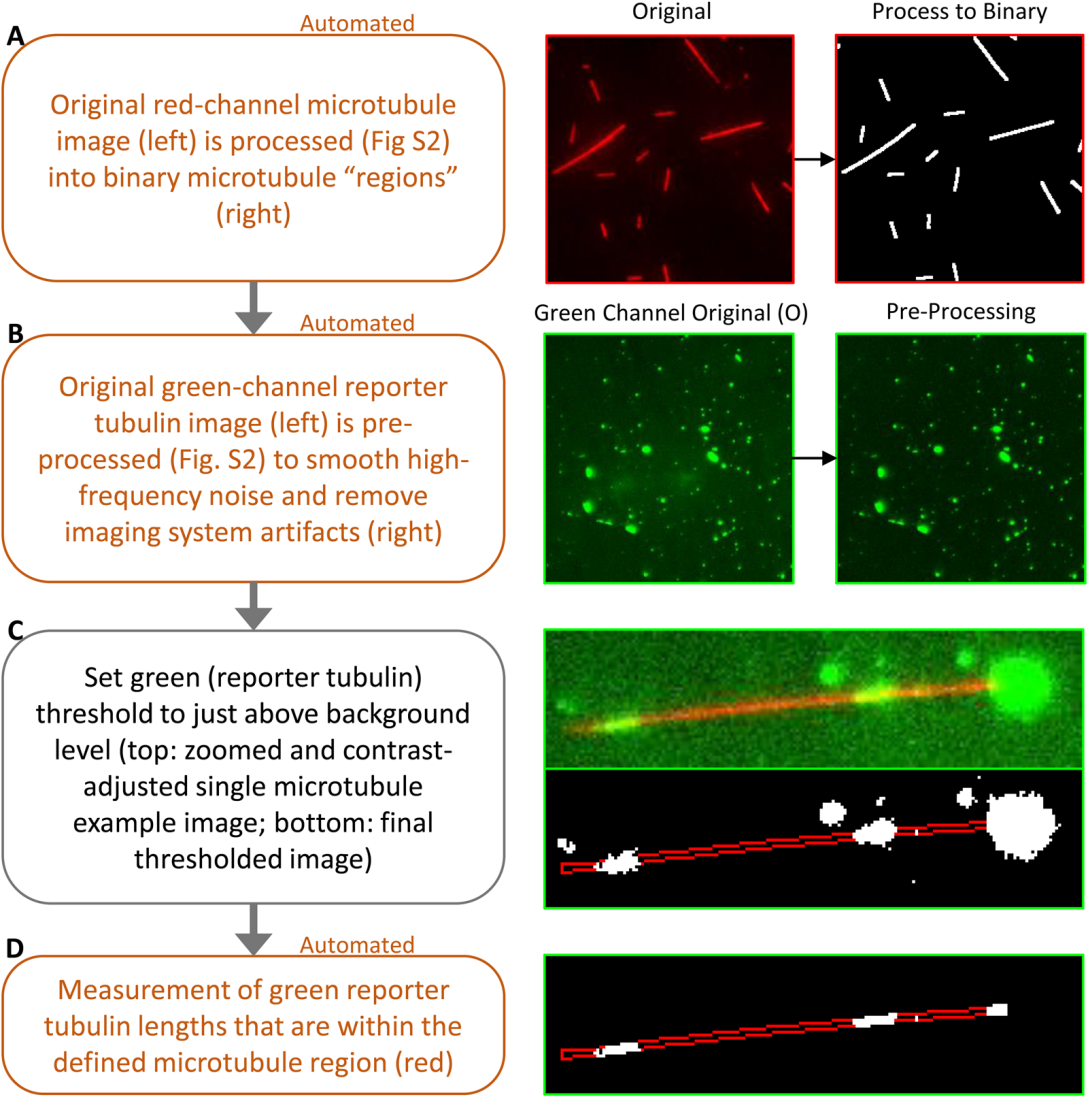
**Automated quantification of gaps in microtubule lattice integrity.** Description (left) and example visualization (right) of the automated TIRF reporter tubulin incorporation analysis assay. A portion of the image in B (right) is shown enlarged in C and D (right).

We then developed a new MATLAB (Mathworks) analysis tool (see Supplementary Materials and Methods) to provide a quantitative measure of the degree of disruption in submicrotubule-scale lattice integrity, as evidenced by the fraction of green reporter tubulin that was incorporated along the length of the red microtubule lattice. This was accomplished first by automatic processing of the red microtubule channel to determine the microtubule-positive regions, which then allowed conversion of the red channel into a binary image with white microtubules and a black background (Fig. 5; extended details in Fig. S2). The green reporter tubulin channel was then also pre-processed to smooth high-frequency noise and to correct for TIRF illumination inhomogeneity (Fig. 5B). The green channel threshold was then manually increased to just above background level (Fig. 5C). The choice of threshold at just above background maintains consistent analysis across multiple experiments while also reliably detecting dim reporter tubulin incorporations into the microtubule (Fig. 5C, right-bottom image represents final thresholded image). Measurements of the reporter tubulin length were then automatically collected from the identified microtubule regions, as indicated by the red outline in Fig. 5D.

**Fig. 6.**
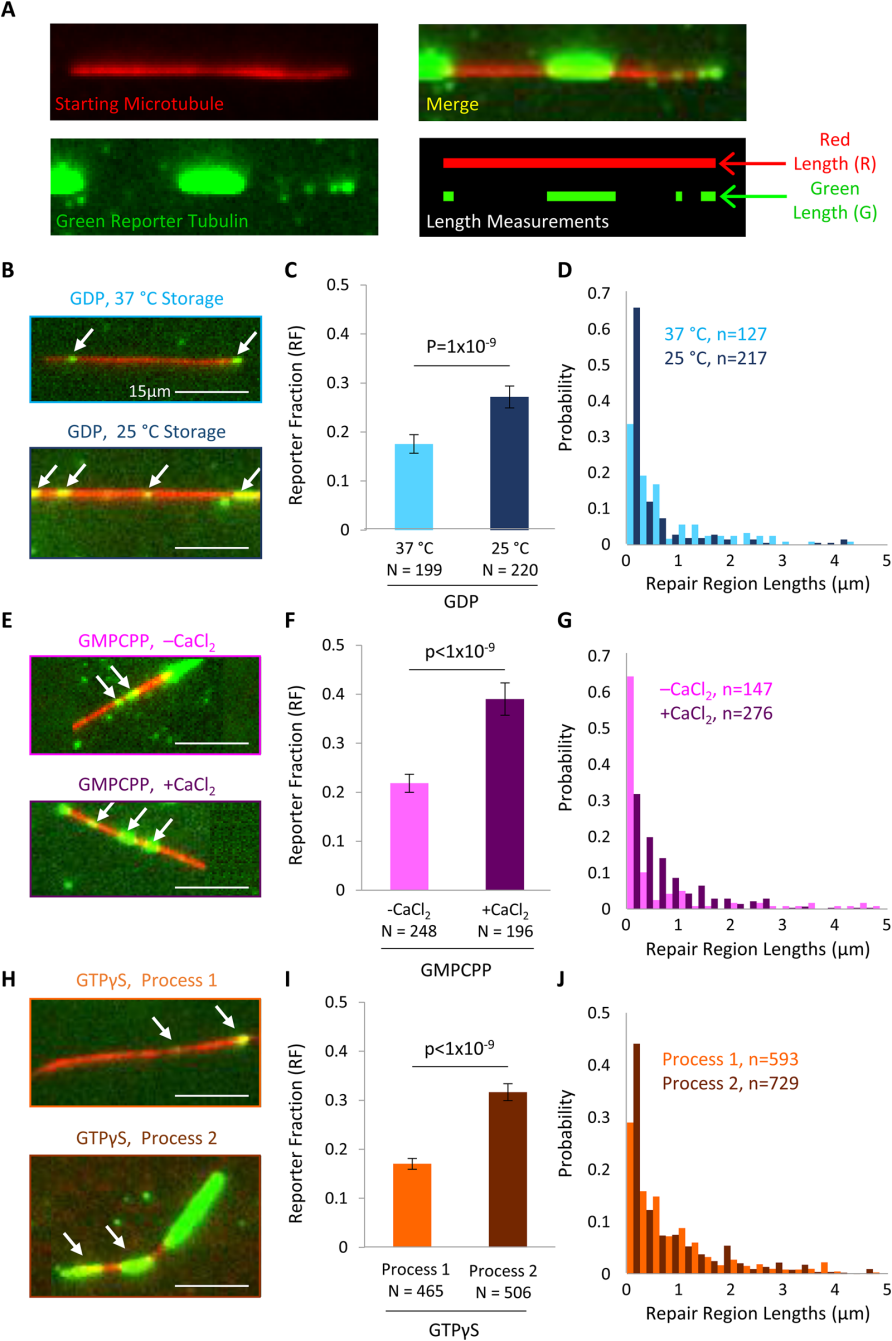
**Microtubule lattice integrity is shifted within microtubule pools.** (A) Top: example microtubule (red) after repair by reporter tubulin (green). Bottom: depiction of quantification technique for Reporter Fraction (RF) using Red Length (R) and Green Length (G). (B) Representative images of microtubules after gap filling assay repair for 37°C storage GDP microtubules (top) and 25°C storage GDP microtubules (bottom). The white arrows indicate sites of reporter tubulin incorporation. (C) The Reporter Fraction is increased for 25°C storage GDP microtubules (right) relative to 37°C storage GDP microtubules, suggesting that storage at 25°C does not promote repair of gaps and defects in the microtubule lattice. (D) Distribution of repair lengths. (E) Representative images of microtubules after gap filling assay repair for untreated GMPCPP microtubules (top) and for GMPCPP microtubules treated with CaCl_2_ (bottom). (F) The Reporter Fraction is increased for CaCl_2_-treated microtubules (right) relative to untreated microtubules, suggesting that CaCl_2_ treatment may lead to gaps and defects in the microtubule lattice. (G) Distribution of repair lengths. (H) Representative images of microtubules after gap filling assay repair for Process #1 GTPγS microtubules (top) and Process #2 GTPγS microtubules (bottom). (I) The Reporter Fraction is increased for Process #2 GTPγS microtubules (right) relative to Process #1 GTPγS microtubules, suggesting that Process #2 does not promote repair of gaps and defects in the microtubule lattice. (J) Distribution of repair lengths. The bar graphs in C, F and I show the mean±s.e.m. repair fraction, weighted by microtubule length (Fig. S3), and corrected for nonspecific background contribution; *P*-values were calculated from Student's *t*-test.

To analyze the degree of lattice disruption for each microtubule, the extent of green tubulin incorporation was quantified by the Reporter Fraction (*RF*). This metric was automatically calculated as the total length of green reporter tubulin signal (*G*; Fig. 6A) divided by the total length of red microtubule signal (*R*; Fig. 6A):(2)
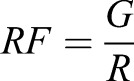

This length-based metric has two key advantages to an intensity-based readout, as (1) it is not sensitive to the variation in image intensity between experiments, and (2) it is not biased by overlap with the green extensions at the growing plus end, which are typically much brighter than most gap-filled sites, and are present on most microtubules regardless of the structural condition. We note that while the repair assay is theoretically sensitive enough to pick up the addition of a single labeled tubulin dimer, our TIRF microscope diffraction limit leads to reporting of the repair length of gaps that are actually significantly smaller than ∼250 nm (even a single reporter tubulin dimer) as repair lengths between 160 nm (our pixel size) and ∼250 nm [diffraction limit, ∼31 dimers in length (250/8)]. Here, the thresholding of the green reporter signal results in some of the dimmer repair regions registering only the brightest pixels in their diffraction pattern, which is why we might detect lengths as low as the pixel size.

To calculate the Reporter Fraction for each microtubule, the red microtubules were automatically detected using the MATLAB script, as described above. False-positives (nonmicrotubules) were deselected manually. For each (red) microtubule, the microtubule length was automatically recorded as the red signal (*R*; Fig. 6A). For each microtubule, the green signal (*G*) was defined as the cumulative length of green reporter tubulin signal that overlapped with the red microtubule, which allowed for exclusion of plus-end extensions (i.e. the green signal that did not overlap with a red microtubule) from the analysis (Fig. 6A). The Reporter Fraction (*RF*) was then calculated as the cumulative length of reporter signal divided by the length of the microtubule (Eqn. 2).

### Results: Microtubule pools have alterations in lattice structural integrity as measured by TIRF Reporter Assay

The TIRF reporter assay was then used to characterize each of our microtubule pools (Fig. 6B-J). First, the lattice integrity of the GDP microtubule pools was evaluated by calculating the Reporter Fraction for each pool. Qualitatively, segments of green tubulin reporter incorporation into the microtubule lattice were more commonly observed in the 25°C storage pool of GDP microtubules than in the 37°C storage pool (Fig. 6B, white arrows). After quantification using the Reporter Fraction (RF), we found that there was a 55% higher Reporter Fraction for the 25°C storage pool GDP microtubules as compared to the 37°C storage pool microtubules (Fig. 6C; *P*<10^−9^), suggesting that the higher overnight storage temperature led to a decrease in defects, gaps, and holes in the microtubules. The mean repair length was similar between the two microtubule pools (Fig. 6D; *P*=0.124, *t*-test), suggesting that the higher Reporter Fraction for the 25°C pool resulted from an increase in the number of repair regions, rather than an increase in repair region length.

Then, the lattice integrity of the GMPCPP microtubules was evaluated by calculating the Reporter Fraction for each pool (Fig. 6E). GMPCPP microtubules treated with CaCl_2_ exhibited ∼80% more incorporation of green reporter tubulin (higher Reporter Fraction) than untreated GMPCPP microtubules (Fig. 6F; *P*<10^−9^). Further, the distribution of repair lengths was shifted upon CaCl_2_ treatment, such that a larger mean repair length was observed for the CaCl_2_-treated microtubules relative to the untreated microtubules (Fig. 6G; *P*<10^−13^, *t*-test). Thus, CaCl_2_ treatment caused the introduction of holes and gaps in the lattice, in addition to the disruptions as were reported by the EM Structure Metric (Fig. 3C).

Finally, the lattice integrity of the GTPγS microtubules was evaluated by calculating the Reporter Fraction for each pool (Fig. 6H). We observed ∼85% more incorporation of green reporter tubulin (higher Reporter Fraction) in the GTPγS microtubules produced by Process #2 as compared to Process #1 (Fig. 6I; *P*<10^−9^). The mean repair length was similar between the two microtubule pools (Fig. 6J; *P*=0.054, *t*-test), suggesting that the increased reporter fraction was due primarily to more reporter repair regions per micron of microtubule. Therefore, by initially growing the GTPγS microtubules at a lower free tubulin concentration, and then by storing them in Taxol under conditions that promoted repair of defects (37°C, higher residual free tubulin concentration in storage solution), this allowed defects in the lattice, such as missing subunits, holes, and gaps, to repair.

### Results: Insight into the microtubule repair process

Finally, we used our new TIRF reporter assay and automated quantification method to dissect a potential mechanism for how the overnight storage temperature, and the associated residual tubulin concentration during storage, may alter the lattice integrity of Taxol-stabilized GDP microtubules.

**Fig. 7.**
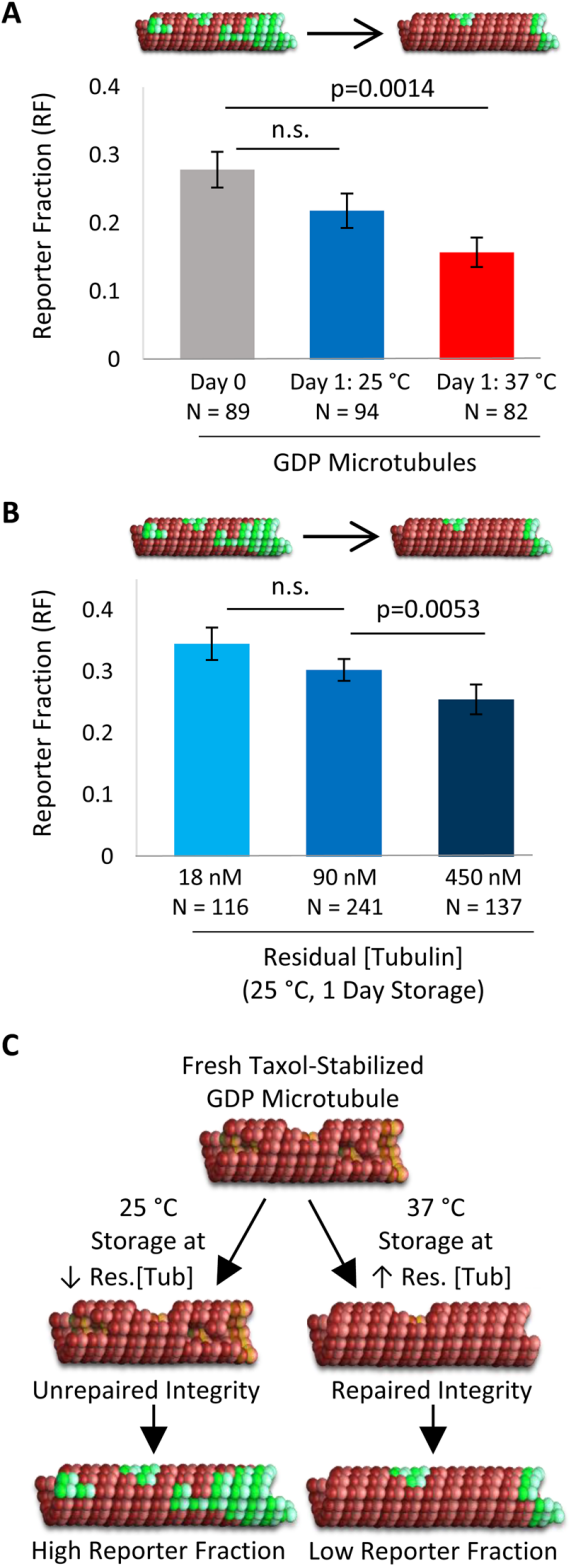
**Impact of storage condition on microtubule lattice structural integrity.** (A) Difference in Reporter Fraction after 1 day storage at 25°C or 37°C. (B) Altered residual free tubulin concentration during storage affects microtubule structure, as shown by Reporter Fraction: increased residual free tubulin leads to a decrease in Reporter Fraction, suggesting that Taxol-stabilized microtubules have fewer defects and gaps when they are stored under conditions of higher residual tubulin concentration. The bar graphs in A and B show the mean±s.e.m. repair fraction, weighted by microtubule length, and corrected for background contribution; *P*-values were calculated from Student's *t*-test. (C) Schematic demonstrating the proposed mechanism for changes in Taxol-stabilized GDP microtubules during storage: 25°C storage and low residual free tubulin concentration prevents repair of damaged microtubules (left, bottom). In contrast, 37°C storage and a higher residual free tubulin concentration lead to repair of damaged microtubules (right, bottom).

To test whether the Taxol-stabilized microtubules were indeed self-repairing their lattice during overnight storage, we compared the Reporter Fraction for newly prepared microtubules as compared to those stored overnight. Here, at Day 0, immediately after the microtubules were prepared and Taxol-stabilized, the microtubule solution was split into two tubes. One tube was stored overnight at 25°C, and the other was stored overnight at 37°C. We observed that the Reporter Fraction was reduced after overnight storage at 37°C (Day 1) as compared to newly prepared, Taxol-stabilized microtubules (Day 0) (Fig. 7A; *P*=0.0014). In contrast, there was a slight but nonsignificant decrease in Reporter Fraction after overnight storage at 25°C (Fig. 7A; *P*=0.160). Thus, newly prepared, Taxol-stabilized GDP microtubules exhibited lattice defects, which were repaired upon overnight storage at 37°C. However, storage at room temperature (25°C) did not facilitate a similar level of repair, suggesting that the storage temperature of Taxol-stabilized *in vitro* microtubules has a significant effect on their structure due to an innate self-repair process.

Because microtubules stored in Taxol solution may repair themselves, even at very low residual tubulin concentrations, we then asked whether changes in the residual tubulin concentration of the storage solution could alter this repair process. In our original GDP-tubulin microtubule preparation, 10 μl of the original polymerized microtubule mixture was diluted into 990 μl warm, 10 μM Taxol Brb80 solution (Fig. 1A, left), which resulted in a measured residual free tubulin concentration in solution of 90 nM. To test whether residual free tubulin could contribute to a repair process during storage, 2, 2.5 and 5 μl of the freshly prepared, polymerized GTP-microtubule solution as described above was diluted into 1000 μl, 250 μl and 100 μl warm Taxol solution (in Brb80), respectively, resulting in residual free tubulin concentrations of 18 nM, 90 nM and 450 nM, respectively. We found that after overnight storage at 25°C, there was a decrease in the Reporter Fraction value with increasing residual free tubulin concentration (Fig. 7B; *P*=0.0053 from 90 nM to 450 nM)). For example, when there was a fivefold increase in residual free tubulin concentration (90 nM to 450 nM), there was a 15% decrease in Reporter Fraction, and, in contrast, when there was a fivefold decrease in residual free tubulin concentration (90 nM to 18 nM), there was a 15% increase in Reporter Fraction, although this change was not statistically significant due to variability in the Reporter Fraction results (*P*=0.249). Additionally, we observed, in a separate experiment, that when there was a 10-fold decrease in residual free tubulin concentration (90 nM to 9 nM), overnight storage at 25°C caused the microtubules to depolymerize completely (data not shown).

Thus, we found that by increasing the residual free tubulin concentration in the overnight storage solution, we could tune the microtubule Reporter Fraction for GDP microtubules, suggestive of a change in microtubule lattice integrity. Consistent with the change in Reporter Fraction from Day 0 to Day 1 (Fig. 7A), this suggests that freshly prepared microtubules, stabilized by Taxol in a single-step process, had disruptions in lattice integrity (Fig. 7C, top), as previously reported (Díaz et al., 1998; Matesanz et al., 2011). A mechanism by which *in vitro* microtubule lattice integrity may be altered during storage is by direct lattice incorporation and repair by free tubulin dimers from solution, especially when stored at warm temperatures (Fig. 7C, bottom). Alternatively, the more disrupted subpopulation of microtubules could also selectively depolymerize during storage. However, since Taxol-stabilized microtubules tended to increase in length as a function of storage time (Fig. S4), it seems likely that damaged microtubules may undergo repair as well.

## DISCUSSION

Through quantification of fluorescence and electron microscopy experiments, we demonstrated that the structural state of microtubules could be manipulated by changes in the growth and storage conditions of those microtubules. These results suggest that the protocols used to prepare, stabilize, and store *in vitro* microtubules can impact the microtubule lattice integrity. This could in turn affect experimental results in studies of motor proteins (Liang et al., 2016) or other microtubule associated proteins (Bechstedt et al., 2014; Davis et al., 2002; Gupta et al., 2013).

It is important to note that our two quantification methods, EM and TIRF microscopy, provide information on different elements of microtubule structural states. The EM Structure Metric (S) reports on the width and curvature of the microtubules, both of which are characteristics of larger-scale changes in the microtubule structural state. The curvature is indicative of more flexibility in the microtubule lattice, which could result from any or all of gaps (Schaedel et al., 2015), unclosed regions of the microtubule (Guesdon et al., 2016), inherent lattice flexibility due to the nucleotide state (Lopez and Valentine, 2014; Valdman et al., 2013; Alushin et al., 2014; Yajima et al., 2012) ,Taxol treatment (Mickey and Howard, 1995; Lopez and Valentine, 2014; Hawkins et al., 2013), or temperature (Kawaguchi and Yamaguchi, 2010). Similarly, increased width is suggestive of an open lattice structure, which may originate from the loss of individual protofilaments, while decreased width is suggestive of holes or gaps in the microtubule lattice.

The TIRF reporter assay is ideal for identifying gaps in the microtubule structure. While gaps and defects can be observed in EM images, the higher throughput nature of TIRF imaging allows for rapid quantification of many hundreds of microtubules for changes in lattice integrity. Additionally, TIRF imaging is sensitive enough to detect repair by a single fluorescent tubulin dimer. Since the Reporter Fraction (*RF*) depends on the relative lengths of red and green fluorescence, it is therefore more sensitive to small gaps than a quantification method based on intensity.

It is important to note that the manipulation of lattice structure for GMPCPP microtubules differed in its implementation and results from that of GDP or GTPγS microtubules. GDP and GTPγS microtubules were initially of low structural integrity as a result of their growth processes and/or the addition of Taxol, and then they were subsequently placed in favorable or unfavorable conditions for repair. In contrast, GMPCPP microtubules were initially characterized by high structural integrity, and were subsequently damaged by the addition of calcium. This resulted in a characteristic difference in the lower structural integrity pool of GMPCPP microtubules as compared to the other two nucleotides in both the EM and TIRF measurements. In the EM measurements, calcium treatment had the distinct phenotype of reducing the median width while increasing the number of curvature outliers. In the TIRF reporter repair assay, GMPCPP calcium treatment was the only condition to shift the repair region length distribution, despite the fact that all three low structural integrity conditions had similar increases in reporter fraction. This is indicative that the method of structural integrity manipulation plays an important role in the resultant characteristics of the microtubule.

Previous work has identified methods for the alteration of microtubule structure through protofilament number control (Bechstedt and Brouhard, 2013). However, the methods and tools described in our new work manipulate a separate element of microtubule structure, namely the lattice integrity, and thus expands the available options for investigations centered on the influence of microtubule structure in microtubule-based cellular processes. The manipulation and quantification of microtubule structure will be useful for future studies focused on the role of microtubule interactions with microtubule-associated proteins.
